# The first three waves of the Covid-19 pandemic hint at a limited genetic repertoire for SARS-CoV-2

**DOI:** 10.1093/femsre/fuac003

**Published:** 2022-01-24

**Authors:** Trudy M Wassenaar, Visanu Wanchai, Gregory Buzard, David W Ussery

**Affiliations:** Molecular Microbiology and Genomics Consultants, Tannenstrasse 7, 55576 Zotzenheim, Germany; Department of Biomedical Informatics, University of Arkansas for Medical Sciences, Little Rock, AR 772205, USA; Retired Scholar, Middletown, MD 21767, USA; Department of Biomedical Informatics, University of Arkansas for Medical Sciences, Little Rock, AR 772205, USA

**Keywords:** SARS-CoV-2, genetic repertoire, mutation frequency, homoplasies, recombination, Pango lineages, variants of concern (VoCs), Omicron

## Abstract

The genomic diversity of SARS-CoV-2 is the result of a relatively low level of spontaneous mutations introduced during viral replication. With millions of SARS-CoV-2 genome sequences now available, we can begin to assess the overall genetic repertoire of this virus. We find that during 2020, there was a global wave of one variant that went largely unnoticed, possibly because its members were divided over several sublineages (B.1.177 and sublineages B.1.177.XX). We collectively call this Janus, and it was eventually replaced by the Alpha (B.1.1.7) variant of concern (VoC), next replaced by Delta (B.1.617.2), which itself might soon be replaced by a fourth pandemic wave consisting of Omicron (B.1.1.529). We observe that splitting up and redefining variant lineages over time, as was the case with Janus and is now happening with Alpha, Delta and Omicron, is not helpful to describe the epidemic waves spreading globally. Only ∼5% of the 30 000 nucleotides of the SARS-CoV-2 genome are found to be variable. We conclude that a fourth wave of the pandemic with the Omicron variant might not be that different from other VoCs, and that we may already have the tools in hand to effectively deal with this new VoC.

## Teaser

With more than 6 million SARS-CoV-2 genomes sequenced, it is possible to look at the mutational frequencies along the mRNA viral genome; only ∼15 loci have significant mutations that stand out above the ‘white noise' background of random mutations.

## Introduction

The SARS-CoV-2 COVID-19 pandemic continues to advance globally. As with every other virus, SARS-CoV-2 viral genomes exist as a fuzzy population, containing mutations that are sporadically being introduced during replication of the RNA genome, along with a subpopulation of defective viral genomes that contribute to the evolution and stability of the virus within a host (Vignuzzi and López 2019). Thus, a viral genome sequence from a clinical isolate represents a snapshot from this noisy background population. When mutations are successfully fixed in the viral population within an individual host and are being transmitted to other individuals, such new virus variants are usually in competition with their progenitors and counterparts. If a given mutation is significantly beneficial in its genomic background (improved fitness), the mutant can outcompete its competitors and become the dominant variant in its locale of transmission. For example, a powerfully beneficial mutation occurred during the early days of the pandemic; it involved the viral Spike (S) protein and resulted in a change of aspartate (D) to glycine (G) at amino acid position 614, designated as S:D614G. This variant soon became overrepresented during the first Covid-19 wave of 2020. The D to G substitution, among several significant benefits, resulted in a more flexible spike-trimer, which improved the binding affinity of S to its primary cellular docking protein ACE2 (angiotensin-converting enzyme 2), enabling more efficient entry into the cell (Ozono *et al*. 2021) and significantly increasing the infectivity of the virus. Because it spread faster than any of its progenitors, the D614G variant became globally dominant by late summer 2020 (Korber *et al*. 2020). As of early December 2021, this mutation is found in >99% of the 6 million viral genomes in the Global Initiative on Sharing All Influenza Data (GISAID) database.

Novel strains with fixed mutations of SARS-CoV-2 have been independently associated with increases in transmission, virulence, immune evasion and therapy resistance (Wang *et al*. 2021 and references therein). As mutations accumulated, more than a thousand individual lineages have now been recognized, with more lineages being added every few days. The lineage nomenclature used here is according to Pango Lineages, which uses the PangoLEARN program to produce phylogenetic trees based on multinomial logistic regression (Rambaut *et al*. 2020).

Of the genetic descendants of S:D614G, multiple variants have been designated as variants of concern (VoCs) because of their higher-than-normal rates of transmission linked to multiple mutations in their S protein. The first variant nominated as a VoC, now named ‘Alpha’ by the World Health Organization (WHO), was initially detected in September 2020 in the South-East of England (Leung *et al*. 2020). The second VoC, ‘Beta’, was first detected in the Eastern Cape Province of South Africa, and the third, ‘Gamma’, was first reported in early January of 2021 in Brazil. Recently, the ‘Delta’ variant, isolated in India in April 2021, has become globally dominant due to its high transmission rate and now represents >90% of the samples sequenced in most countries. The other proposed VoCs and variants of interest seem to be outcompeted by Delta in most regions. However, at the time of writing, a new variant, Omicron (B.1.1.529), is predicted to soon be the dominant VoC in some countries in Europe.

The nomenclature for VoCs, proposed by the WHO to provide geographically neutral descriptors, is somewhat confusing in relation to viral taxonomy, which also uses Greek letters for defining some viral genera, including the members of the coronavirus family. Technically, Coronaviruses are a subfamily of *Orthocoronavirinae*, and they are divided into four genera: Alpha-, Beta-, Gamma- and Deltacoronavirus. SARS-CoV-2 is the second outbreak strain of the SARS-CoV species, which is a member of the genus of Betacoronavirus; the Alphacoronavirus genus also contains two common cold coronaviruses that can infect humans (viruses from the other two genera, Gammacoronavirus and Deltacoronavirus, mainly infect birds). Despite this, the WHO nomenclature for SARS-CoV-2 VoCs has been adopted globally. However, with the increasing use of whole or partial genomic sequencing, other potential VoCs will eventually appear, and the Greek alphabet is likely to run out of letters to describe them.

The original lineage nomenclature describes Pango Lineages by a combination of a letter (or, for later lineages, two letters) and one or more numbers (Rambaut *et al*. 2020). By that nomenclature, Alpha is known as lineage B.1.1.7, Beta is B.1.351 (it was originally named 501Y.V2 after a mutation in its spike protein) and Gamma is lineage P.1. The variant that caused the large wave that swept through India in the first half of 2021 was originally designated B.1.617, but that was soon split up into sublineages, of which B.1.617.2 was subsequently designated as Delta. The recently identified Omicron variant (B.1.1.529) was first characterized in South Africa in November 2021 and it is quickly spreading across the world.

Alpha was initially able to spread more rapidly than other variants locally present across the globe, and toward the end of 2020 it became dominant in most of Europe (Davies *et al*. 2021). Its transmissibility was estimated to be 1.5 times higher than that of other circulating lineages in the UK at that time (Vöhringer *et al*. 2020), likely because of its spike protein mutations. Artificially introduced mutations have been reported to enhance its affinity to the ACE2 receptor by 35-fold *in vitro* (Chan *et al*. 2021).

Since their discoveries, Beta, Gamma, Delta and now Omicron have been detected in multiple countries, but so far only the latter two have been spreading faster than existing endemic variants in most of the geographic regions where they were detected. Recent results indicate that viral loads can be >1200-fold higher in patients with the Delta variant than those observed with other lineages (Li *et al*. 2021). One of the reasons VoCs are of ‘concern’ is because of their many changes in the Spike protein, which can potentially allow the variants to become immuno-escapees by reducing acquired host immune-protections gained from either a natural SARS-CoV-2 infection, from the current Spike vaccines (Chen *et al*. 2021; Garcia-Beltran *et al*. 2021), or from monoclonal or convalescent sera therapies. However, which mutations are responsible for this, how many in combination in the S protein are critical and what other mutations are required for higher infectivity and virulence remain unclear.

Even though the VoCs are considered to represent well-defined variants, their genomes are not stable over time, as they continue to evolve. Every single viral lineage represents a heterogeneous population of genomes, a fact that is not always clearly acknowledged. Phylogenetics within SARS-CoV-2 is complex, as identical mutations known as ‘homoplasies’ have independently arisen in various branches, and the phylogenetic trees typically contain ‘polytomies’, with multiple branches originating from a single node where a branching order cannot be determined. It was recognized early on during the epidemic that homoplasies were frequently found in SARS-CoV-2 sequences (De Maio *et al*. 2020). Several authors have suggested that genomic recombination between different lineages, clades or variants of SARS-CoV-2 may have occurred to account for the homoplasies (Korber *et al*. 2020; Jackson *et al*. 2021; Taghizadeh *et al*. 2021; Varabyou *et al*. 2021; Vasilarou *et al*. 2021). A role for NSP14, the proofreading exoribonuclease of this virus that accounts for its unusually high replication fidelity, has been proposed in these postulated recombination events (Gribble *et al*. 2021). However, homoplasies can also result from recurrent mutations (parallel evolution) occurring in mutational hotspots, with or without convergent positive selection (Tonkin-Hill *et al*. 2021). In addition, disadvantageous mutations can revert to the original sequence, which further confuses phylogenetic analyses.

Here, we compare a set of time points, starting with a large set (over 400 000) of SARS-CoV-2 genomes that were downloaded from the GISAID repository in February 2020, for which we have identified conserved mutations within each of the given Pango lineages. We then screened how frequent those lineage-conserved mutations were observed in every other lineage. We consider this a valuable addition to previously published phylogenic-centered analyses. As a follow-up, when more than a million Delta genomes had been sequenced and deposited, these were separately analyzed. Finally, we briefly compare our results with the as yet limited number of Omicron genomes available to us.

Our aim is not to provide an extensive review of the available literature, but rather to investigate the mutational behavior of SARS-CoV-2, based on deposited genome sequences. Before presenting the results of our investigation, we briefly discuss the sequence repositories that collect the genomes of this virus and on genome sequence quality.

## Repositories of sars-CoV-2 genomes in view of the fair requirements and data quality

Traditionally, publicly shared nucleotide and protein sequences are submitted to GenBank, and/or EMBL or DDBJ, the two other sequence repositories that are synchronized daily with GenBank. In these databases, sequences can be searched and retrieved, together with their metadata. GenBank fulfills the criteria for scientific data management and stewardship that are abbreviated as the acronym FAIR: Findability, Accessibility, Interoperability and Reusability (Wilkinson *et al*. 2016). The earliest available genome sequences of what was then called 2019-n-CoV were submitted to GenBank and were used by the international research community for the development of detection technologies and vaccine design. Currently, there are close to three million SARS-CoV-2 genomes deposited in GenBank.

For SARS-CoV-2 genomes, there is a very large and highly popular alternative sequence repository—the GISAID. This platform was originally set up for the rapid exchange of outbreak data (Elbe and Bickland-Merrett 2017) and it was adapted for the storage of SARS-CoV-2 genomic data. Although the genomes stored in GISAID are shared with researchers, technically they are not part of the public domain, as access is restricted by login, and the submitting authors retain the rights to their data. In early December 2021, GISAID contained over 6 million SARS-CoV-2 genomes, more than twice as many as in GenBank. Currently, there is substantial overlap between these two repositories, but they are not exact copies.

In GISAID, the Pango lineages of all deposited genomes are recorded and are easily searchable. Searches can also be limited to the geographical origin or collection date, or by the presence of a given amino acid mutation, or by VoC, which are all highly useful functions. GenBank now has a separate Data Hub for SARS-CoV-2 as part of NCBI (https://www.ncbi.nlm.nih.gov/sars-cov-2) where Pango Lineages can also be searched.

A problem is that Pango lineages are being redefined over time, and can be renamed, split up or combined, sometimes several times within a month. There is no transparency as to which genomes that were once attributed to one lineage, later ended up in another, and this severely limits the reusability of data over time. All deposited genomes have a database-specific unique identifier (UI), which in GenBank is its INSDC accession number (which is the same in the EMBL and DDBJ databases); in GISAID, the accession identifier starts with the letters ‘EPI’. The UI of GenBank and GISAID are not reciprocally recognized, thus, there is no easy way to tell whether a genome sequence of a single isolate has been uniquely deposited in GISAID, in GenBank, or in both. We estimate that ∼20% of the SARS-CoV-2 genome sequences in GenBank are absent in GISAID, and more than half of the 6 million GISAID sequences are not represented in GenBank. Combining the two datasets would produce a large degree of redundancy that is tedious to remove. This hampers interoperability. Lastly, the search options in GISAID are limited, for instance, a Boolean command ‘NOT’ is not available to exclude certain search terms. Following a selected search, retrieved records are presented in pages of 50 entries only. On the other hand, searching for sequences belonging to a given Pango lineage, isolated in each geographical region, or during a given time, is difficult in GenBank (although this utility is improved on the SARS-CoV-2 Data Hub). In conclusion, both databases have advantages and limitations, so neither is perfect.

It is important to note that both databases suffer from entries of genome sequences that are highly incomplete or of poor quality. This is mainly due to the practice of sequencing the viral genome by sequencing technologies that produce short reads averaging ∼200 nucleotides, following PCR amplification using a large set of PCR primers. If a given set of primers fails to amplify its targeted genomic region, the predicted length of those missing sequences is given as N-stretches, which is considered acceptable if ‘essential’ regions of the genome are covered by the deposited sequences. In GISAID, sequences are defined as ‘complete’ if they are at least 29 000 nucleotides long, as ‘high-coverage’ if they contain <1% Ns, and as ‘low-coverage’ if they contain >5% Ns. By these definitions, of the 6 million genomes in GISAID at the time of this writing, 5.9 million are ‘complete’, 4.3 million (∼73%) have ‘high-coverage’, and 17% have ‘low-coverage’. Entries of ‘low-coverage’ can contain as many as 30% nondefined Ns. The applied quality filter of <1% Ns is not particularly strict, as it would still allow for 300 undefined nucleotides in the 30 000-nt genome, and most of the genome sequences in GISAID contain a few hundred ‘Ns’. For bacterial genome sequences, quality scores have been defined that put a penalty to any stretch of Ns longer than 9; for viral sequences the length of SARS-CoV-2, we recommend stretches of 5 Ns to be applied as quality score as the upper limit to identify a high-quality genome. But if one of those five ‘Ns’ happens to be in an important region that determines a VoC, this can still cause problems.

## How to assess the genetic repertoire of over 400 000 SARS-CoV-2 genome sequences

A ‘standard’ way to compare genomic sequences is by multiple alignments, from which a phylogenetic tree can be produced, making use of dedicated algorithms that can predict the most likely phylogenetic relationship of highly similar sequences. Alternatively, a cladogram can be created that simply bins the sequences into groups based on their similarities. However, for larger datasets, this can become difficult to visualize. We followed a slightly different approach: we started with the Pango lineages to which genomes have been attributed, as defined by the GISAID database. As discussed above, Pango Lineages are not completely static, so the analysis represents a snapshot of a moment in time only, but since Pango Lineages are being used by many scholars as one way to identify to which group a given genome belongs, we considered this a valid approach.

Our first download of genomes from GISAID for analysis was performed on the first of February 2021, when Delta was rarely found and not yet designated as a VoC. We used those downloaded genomes to clarify which of three possible mutational events—recombination, parallel evolution or reversion—is disrupting the apparent linear phylogenetic relationships within SARS-CoV-2. For this, we recorded the presence of consistent mutations relative to the Wuhan reference sequence. In contrast to what is commonly done, we also included all nucleotide changes in the genome, including those that do not affect the viral proteins; first, because they convey important phylogenetic information, and second, because they may affect RNA folding or other regulatory mechanisms. Together with the positions involved in interactions with the structural Nucleocapsid (N) and Membrane (M) proteins for genome packing, these mutations may be under a variety of selective pressures. Following the downloading of 450 968 genomes from the GISAID database, the quality assessment was applied to remove genomes that contained N-stretches longer than 5, which resulted in a cleaned set representing 90% (410 379) of the deposited genomes. These quality-controlled genomes were binned according to their Pango Lineage, as recorded on the day of the download; genomes without an assigned Pango Lineage were excluded. This step maintained 410 171 genomes from 866 Pango Lineages. Note, these defined Pango Lineages are not stable over time, as will be discussed below.

For each Pango Lineage recognized in February 2021, we defined all mutations that were present in at least 95% of its members. These were mapped to the reference Wuhan-Hu-1 SARS-CoV-2 genome (GenBank accession MN908947.3; for extensive annotation, see NCBI reference sequence NC_045512.2) that we herein call Wuhan V_0_. This reference was used to identify the lineage consensus nucleotides, one lineage at a time, to create 866 artificial lineage consensus sequences (LCS). This step significantly reduced the mutational noise generated by spontaneous isolate-specific variations. Although not all members of a given Pango Lineage are identical, nearly all members (to be precise, >95% in our case) of a given lineage share at least the Pango Lineage-conserved mutations, and those were used here to create the LCS. These were then used for several informative comparisons presented below. The use of LCS that cover all mutations conserved at 95% within a lineage means that temporary and local mutations resulting from ongoing genetic drift are ignored. We further point out that bias in sampling and sequencing practices may have eliminated mutations that are underrepresented in the available data. We did not concentrate on the frequency of transitions versus transversions, or synonymous versus nonsynonymous mutations, as such comparisons have been done extensively by others (e.g. Tasakis *et al*.^2021^; Tonkin-Hill *et al*. 2021).

## Mutational frequency along the SARS-CoV-2 genome

**Figure 1.**
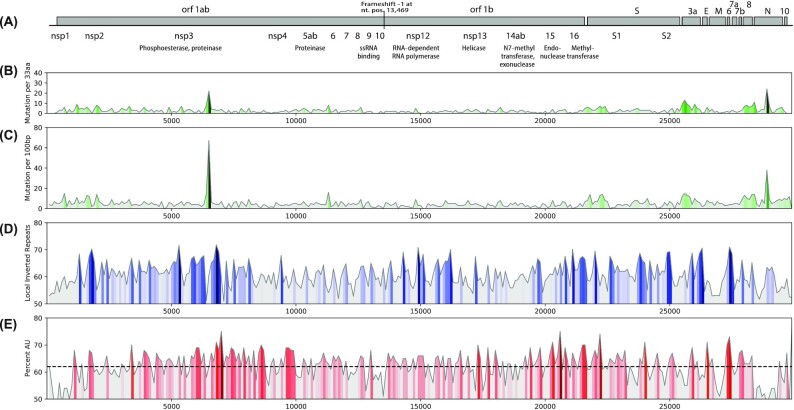
Mutation density and genomic features of Sars-CoV-2. Positions of open reading frames are shown in panel **(A)**. The frequencies of amino acid changes **(B)** and nucleotide mutations **(C)** are colored with light grey to dark green for low to high mutation frequencies, respectively. Panel **(D)** shows the percentage of local inverted repeats in the Wuhan-Hu-1 reference genome, which can be used as a proxy for regions likely to form stable stem-loop structures (Jensen, Friis and Ussery 1999), indicated by the dark blue peaks. Panel **(E)** shows the average AU content along the Wuhan-Hu-1 chromosome for a window of 100 nt. The average %AU is represented by a dashed line.

From the downloaded dataset of 1 February 2021, a total of 1462 nucleotide positions were identified that had changed compared with the reference, representing roughly 5% of the genome. These represented a total of 900 changes in amino acid sequences. For a first assessment, the 866 LCS were used to map the frequency of amino acid and nucleotide mutations along the genome (Fig. 1). This identified three regions that displayed a relatively high mutation density. A sharp peak is visible around nucleotide 6330 in open reading frame 1ab (Orf1ab) positioned around amino acid 1200 of nonstructural protein 3 (NSP3). A second region is found in the N-terminal region of S, and a third region is located within the Nucleoprotein gene. The mutation frequencies in NSP3, S and N were higher for nonsynonymous mutations than for synonymous mutations, indicative of selective advantage pressure on the proteins they code for. Recently, single nucleotide variants were mapped using a 250-nucleotide window along the SARS-CoV-2 genome, which resulted in several hotspots (Mandal, Roychowdhury and Bhattacharya 2021); however, the reported locations of those hotspots did not overlap with the regions identified here as collecting mutations at high frequency. This may be because of the small sample size of the SARS-CoV-2 genomes analyzed by Mandal and colleagues (617 genomes only, compared with over 400 000 here) and by their inclusion of MERS and SARS-1 genome sequences. Figure 1 also shows the presence of local inverted repeats, as a proxy for stem-loop structures, which can stabilize secondary structures in the single-stranded mRNA genome; there is a strong peak around nucleotide 6400 in Orf1ab that is particularly AU rich (Fig. 1D and E). A correlation between AU content and mutation frequency was not observed.

## A matrix captures all mutations in the 688 pango lineages and identifies a limited genetic repertoire

**Figure 2.**
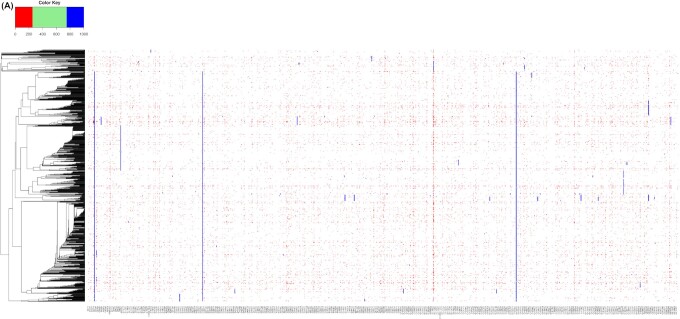

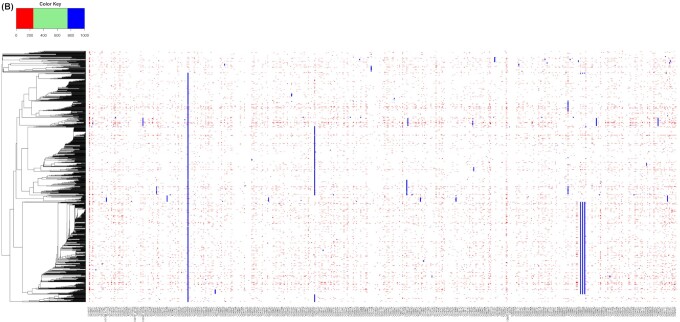
Matrix of all 1492 lineage-conserved (>95%) nucleotide mutations recorded in 410 171 SARS-CoV-2 genomes that are conserved in at least one Pango lineage at >95%, reporting their presence in any other lineages at >0.5%. The 688 lineages are clustered based on all recorded mutations, including synonymous substitutions and deletions. Panel **(A)** shows the first 900 variable positions covering orf1ab and orfb, and panel **(B)** the remaining 592. The lineage names listed to the right are not complete and are only indicative. The positions of B.1.1.7 and B.1.351 are indicated by black arrows (P.1 is positioned directly above B.1.1.7). Three groups of lineages with multiple sublineages that mostly contain identical conserved mutations are also indicated. Major mutations occurring in multiple lineages are indicated inside the matrix. Capitals are used for amino acid substitutions (numbered for the amino acid position in the spliced protein) and lower case letters for synonymous nucleotide changes, numbered for the position in the reference genome, NC_045512.2. A position that flips between two nucleotides at low frequencies in multiple lineages is indicated by an arrow at the bottom of panel (A). The black square with dotted lines inside panel (A) indicates the position of the zoom shown in Fig. 3. Two homoplastic mutations, Q57H and S194L, are indicated in panel (B) with light-blue boxes.

Every Pango lineage contains at least one strongly conserved mutation or a unique combination of mutations. However, a significant number of these lineage-conserved mutations can also be found in other lineages. This is to be expected, as a lineage is derived from an ancestor, thus descendants will normally bear all the mutations that were already present in their ancestral lineage. We determined the frequency of each lineage-specific mutation in members of all other lineages, and visualized the results in a large matrix, split up into two panels for a graphical representation (Fig. 2). The columns of the two matrix panels combined represent all 1462 nucleotides of a conserved mutation found in at least one Pango lineage, with 900 such positions in Fig. 2A and 592 in Fig. 2B. These mutated nucleotides were the ‘queries’ to score their presence in every other lineage. The rows of the matrix panels are formed by the 866 Pango lineages that were defined at the time of download. These were ordered by unweighted cluster analysis of all mutations considered, giving a matrix of over 1.26 million cells (panels 2A and 2B combined). A matrix cell is colored if the query mutation was present in at least 0.5% of that lineage's members. This requirement was not met for 96.7% of the cells, so they remain white. The others were colored, with red for low frequencies (<25%) for a given mutation in a given lineage, blue for high frequencies (>75%) and green for frequencies between these limits. Although there are many white cells, only 75 mutations were completely lineage specific, as they did not reach frequencies above 0.5% in any other lineage. A striking observation is the large number of red cells in the matrix, suggesting that a mutation found at 95% conservation in each lineage (which is the requirement to be included in this matrix) is often found at a low frequency in one or more other lineages. A second noteworthy observation is that mutations are either found at high frequencies (>75%) in a lineage (in total, 7762 cells in the matrix are red) or at low frequencies (<25% for 33 879 cells shown in blue), but relatively few (only 474) are found at frequencies between 25% and 75%. This provides us with the first hint that a given mutation can be maintained in one lineage (hence, it is captured in the matrix in the first place) but when introduced in another genetic background, it may not be advantageous, and if sufficiently disadvantageous, it can be subject to purifying selection resulting in reversal or removal.

Since the matrix of Fig. 2 shows all observed mutations aligned next to each other, the peaks and troughs visible in Fig. 1 that were caused by variation in mutation densities along the genome are not apparent in the matrix. Because of the scale of the matrix, individual cells are barely visible, unless mutations are present in multiple neighboring lineages: in that case, they form vertical lines. Indeed, a number of vertical lines are visible indicating highly conserved mutations, some of which are pointed out in the figure. In the figure, and throughout the text, uracil is written as T, and synonymous mutations and those occurring in noncoding regions are indicated by lower case letters and are numbered according to their nucleotide position in the reference genome. Nonsynonymous mutations are given as amino acid substitutions, in capitals, and these are numbered according to the protein in which they reside in the reference genome.

The widespread occurrence of the NSP12:P323L in ORF1b (RNA-dependent RNA polymerase, RdRp) is clearly visible as a line running through most of the Pango lineages and that mutation is conserved together with S:D614G and with two synonymous mutations in the nontranscribed 5′-region (c241t and c3037t). These four mutations arose at a very early stage in the pandemic and have been retained in their subsequent offspring. Two other widely represented mutation events involved a three-nucleotide change in the N gene resulting in amino acid changes N:R203K/G204R, typical for members of the lower cluster, and ORF2a:Q57H, which occurs in lineages in the middle cluster of Fig. 2B.

As would be expected, some mutations are conserved in combinations between multiple lineages, and a number of these are visible as short vertical lines in the figure. However, their appearance is affected by the way the various sublineages were defined at the time of download. For example, the group of B.1.258 and its sublineages B.1.258.XX mostly contain the same set of mutations, and these form multiple vertical short lines in this part of the matrix; this is also observed for the groups of B.1.177 or B.1.36 and their sublineages. These groups are indicated to the right of the matrix in Fig. 2.

The key message that Fig. 2 conveys is that the genetic repertoire of SARS-CoV-2 seems to be limited. A mutation that is found conserved in one lineage is often also found in other, nonrelated lineages, where it must have evolved independently and is found at low frequency. This suggests that these mutations occur spontaneously and repeatedly. Mutations that have arisen for the first time during the later phases of the pandemic are relatively uncommon, a point we will elaborate on.

## Homoplasies are common between lineages of SARS-CoV-2

**Figure 3.**
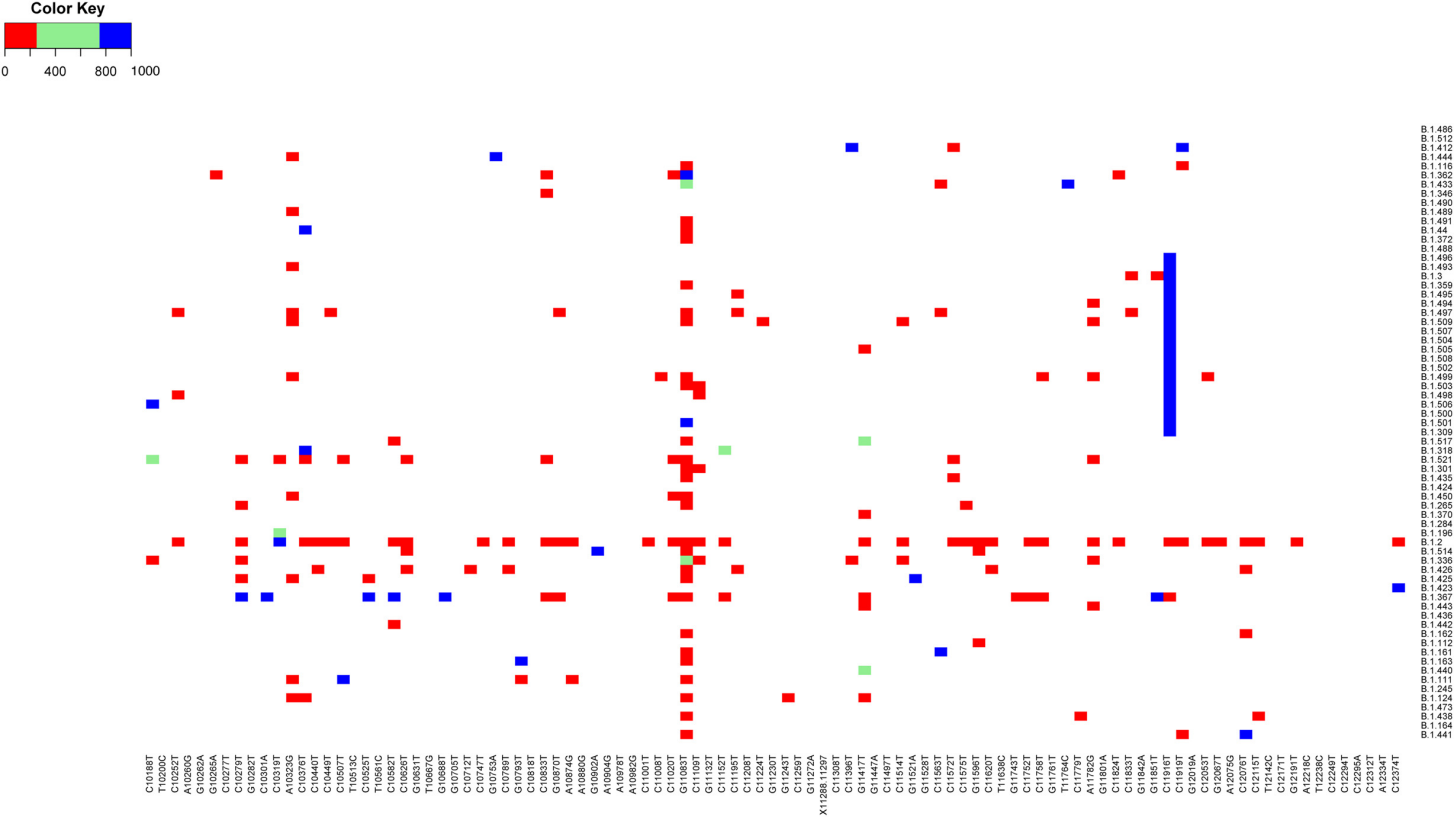
Zoom of the matrix for a region in orf1ab. All mutations and lineages included in this zoom are now labeled and visible on the *X* and*Y* axes, respectively. Two positions indicative of homoplasies are indicated by light-blue boxes. Mutation g11083t leading to NSP6:L37F varies between wild type and mutation at variable frequencies, from 0.5% to 100%. Mutation c1916t resulting in NSP7:S25L is conserved in a number of lineages and also found at low frequency in two unrelated lineages.

Figure 2B shows that the mutation ORF3a:Q57H is found not only in all members of the middle cluster but also in a number of genomes at the very bottom of the matrix (indicated by a light-blue box in panel 2B). The same applies to N:S194L, which is shared by members of two different major clusters; these are just two examples of homoplasies. A homoplasy is a ‘characteristic’ (here, a mutational event) that has been gained or lost independently in separate lineages over the course of evolution, and that cannot be parsimoniously explained by descent from a common ancestor. In fact, many more examples exist of identical mutations recorded in lineages that are not clustered together in the matrix, strongly indicative of homoplasies. Most homoplastic mutations are found at low frequencies in lineages (red-colored cells in Fig. 2), but in some cases, a homoplasy is conserved in two nonrelated lineages. This is better visible in Fig. 3, where a zoomed fragment of the big matrix of Fig. 2A is displayed that covers mutations located in Orf1a (in NSP5 to NSP8). Several homoplasies are indicated by light-blue squares in the figure. The mutation NSP7:S25L is pointed out as an example, which is conserved in 20 lineages, but it is also found at low frequency in two members of a different cluster. One of the lineages containing S25L at high frequency (B.1.506) also contains c10188t in 97% of its members, as indicated by a light-blue box to the left of the figure. That synonymous mutation is also found in 69% of members of B.1.521 in a cluster below that of B.1.506, which, however, does not contain S25L. Likewise, mutation NSP5:P108S is found in lineages B.318 (at 100%) and in B.1.44 (at 98%). Nevertheless, because of mutations elsewhere in their genomes, these lineages are not clustered together in the matrix, so their common mutations represent homoplasies.

The identification of homoplasies in this manner strongly depends on the clustering of the genomes. The clustering shown in Figs 2 and 3 is based on all mutations reaching >5% of lineage members to maximize resolution. We checked to see if alternative clustering could reduce the apparent number of homoplasies. For this, the Pango Lineage consensus sequences were used. Neither MASH clustering nor phylogenetic clustering resulted in trees resulting in higher resolution or fewer homoplasies.

The possibility that lineage-specific mutations that are observed in other lineages at low frequencies were caused by inaccurate lineage attribution was considered, but that explanation could be rejected, as for each lineage investigated, the lineage-specific mutation(s) resulted in frequencies close to 100%. If inaccurate lineage attribution were at the heart of the observations reported here, those lineage-specific mutations should have been observed at lower frequencies as well. We conclude that homoplasies are widespread in this virus.

## Reversions of mutations are observed, sometimes at high frequency

Figures 2 and 3 identify a few nucleotide positions that appear to alternate between two nucleotides in many lineages. The starkest example is mutation g11083t, responsible for NSP6:L37F, which has become fixed in several lineages but forms a nearly continuous red line in panel 2A. Possibly, this and other regularly alternating mutations are not only introduced but are also reverting back to wild type at a high frequency. To investigate the possibility of reversion in more detail, we concentrated on the four mutations that had been introduced very early during the epidemic. Although their presence was recorded in nearly all lineages that evolved since B.1 appeared (the exception being B.1.14, which lacked all four), not all members of those offspring B.1 lineages contained all four of the mutations at the same time. Table S1 (Supporting Information) summarizes 21 sublineages of B.1 that lack one or more of these mutations in >5% of their members, most likely because of reversions. For many of the listed lineages, all their members contain two or three of these four mutations (at 100% conservation), but the remaining mutation(s) is/are only found in a fraction of their members. The table also includes lineage A.10, which contains S:D614G but not c241T or NSP12:P323L, while the synonymous c3037t mutation is found in 38% of its members (Table S1, Supporting Information). For several of the listed lineages in the table, members were found containing wild-type or mutation sequences in variable combinations. This is difficult to explain by individual recombination events that had occurred with a genome of a lineage lacking these mutations; more likely, the observations suggest that these mutated positions can and do revert to wild type at variable frequencies.

## Homoplasies are often the result of parallel evolution

**Figure 4.**
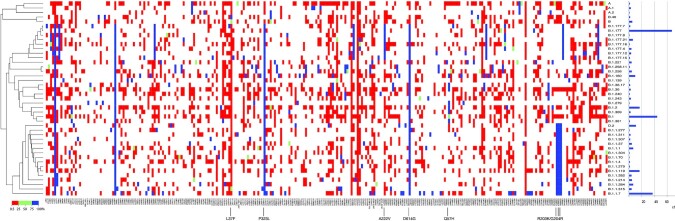
Matrix of mutation frequencies in the 44 Pango lineages that were represented by >1000 genomes in the GISAID database on 1 February 2021. The number of genomes for each of these is shown in the histogram, to the right of the matrix. The conservation of multiple mutations in various lineages, often at low frequency (red), is clearly visible.

The matrix in Fig. 2 represents all recognized Pango lineages as defined in February 2021, but some of these were more successful spreaders than others during the early phase of the pandemic. A smaller matrix is shown in Fig. 4, now only containing the 44 Pango lineages that were represented by >1000 genomes each in the cleaned dataset of 1 February 2021. These collectively represent nearly three-fourths (74%) of all genomes of that dataset. Although the downloaded sequenced genomes may not truly represent the success of individual lineages during the pandemic, due to sampling and sequencing bias, it provides a rough indication of lineages that were particularly successful in propagation. A striking observation from Fig. 4 is that mutations are either found at low frequency within a given highly successful lineage (colored red) or strongly conserved in that lineage (colored blue) but very few positions report mutations at a frequency between 20% and 80%. This indicates a strong purification selection even in highly successful clones.

Lineage B.1.177 (not to be confused with B.1.1.7, which is Alpha) was the most abundant lineage in the dataset at the time (17%). The combination of 11 mutations that define B.1.177 are also conserved in all of its offspring (B.1.177.1 to B.1.177.27, as they were called in February 2021) and these form short vertical lines in the matrix of Fig. 2. Of the sublineages of B.1.177, seven were represented by >1000 genomes, and if these had all been combined with B.1.177 in one lineage, they would collectively have represented 21% of all genomes in our downloaded dataset. Some of the homoplasies observed in B.1.177 members are illustrated in Fig. S1 (Supporting Information).

The second most abundant lineage was B.1 (11%), but apart from the conserved synonymous mutations c241t and c3037t, combined with S:D614G and NSP12:P323L, its members did not consistently contain other mutations. The third most frequently sequenced Pango Lineage was Alpha, which was well on its way to growing in abundance at that time. Homoplasies were identified in many of these successful lineages, of which some are summarized in Table S2 (Supporting Information). Thus, homoplasies can be found in highly successful lineages, indicating they can contribute to the fitness success of a lineage.

Table S2 (Supporting Information) contains examples of homoplastic deletions in the VoC Alpha, Beta and Gamma. Since deletions cannot easily revert back to wild type, the likely explanations for homoplastic deletions are either recombination or parallel evolution. We separately analyzed all detected deletions in the complete dataset. Eight deletion events were completely lineage specific, as these were only found in one lineage each. One deletion was found in 11 lineages, but it was specific for these lineages only, and those containing them did not contain other deletions. A further deletion, removing nine nucleotides from NSP1 (K141-/S142-/F143-), was observed at low frequency in multiple lineages. It can be found in 100% of members of one lineage (B.1.372) but also at frequencies below 10% in others, while a total of 685 lineages lacked this deletion completely. Low frequencies of this deletion within individual lineages suggest the deletion was introduced independently and repeatedly and it was not present in the founder of that lineage.

Low frequencies of recurrent mutations in SARS-Cov-2 have been reported before, based on smaller datasets, e.g. by Alouane and colleagues, who compared 30 000 genomes (Alouane *et al*. 2020). A similar approach to our analysis was recently published, where the authors concentrated on spatial-temporal trends, based on a clustering of genomes into ‘macro haplotypes’ similar in concept to multilocus sequence typing (Chiara *et al*. 2021). These authors did not point out the homoplasies that were recognizable in their data and only proposed recombination events to have occurred prior to the jump of SARS-CoV-2 to the human host.

Multiple authors have proposed that recombination may have occurred during this pandemic. Undoubtedly, evolutionary-level recombination in other Coronaviruses are common and they may even have been responsible for formation of a SARS-CoV-2 ancestor prior to its jump to the human (Dhama *et al*. 2020; Flores-Alanis *et al*. 2020; Zhu, Meng and Meng 2020; Singh and Yi 2021). It is also possible that recombination between different species of Coronaviruses have occurred in an intermediate host between humans and bats, in case that scenario took place, as pointed out by others (Boni *et al*. 2021). Some authors have proposed that recombination events were also responsible for the formation of novel lineages within SARS-CoV-2, as two viral variants co-propagated in human individuals (Van Insberghe *et al*. 2021). Note that homologous recombination between the core RNA and the transcription regulating sequence (TRS) can also result in mutations, as was suggested to have produced N:203K/G204R (Leary *et al*. 2021), and that possibility is not rejected here. As recombinations producing chimeric genomes require a simultaneous double infection with two variants, such an event is more likely to occur in regions where different variants are present at high incidence. Optimal conditions for inter-genome recombination occurred in the UK between October 2020 and January 2021, when both Alpha and B.1.177 members were abundant (Jackson *et al*. 2021). When specifically searching for chimeric genomes that would have arisen from recombination events between these two, these authors identified 16 examples out of 279 000 genomes that had been isolated in that period (Jackson *et al*. 2021). One of these circulated for 9 weeks and resulted in at least 45 infections. This suggests that recombination can occur during human double infection, but it seems to take place at a low frequency only and have minimal fitness advantage.

There also seem to be constraints in fitness when combining mutations of Alpha with mutations of another lineage. However, as we point out here, the number of homoplasies amongst SARS-CoV-2 genomes is extensive, not only for mutations observed in the majority of genomes belonging to particular Pango lineages, but also when considering mutations observed at low frequencies within the lineages. It is virtually impossible to explain this extent of homoplasies by recombination events that seem to occur only under specific conditions (multiple variants infecting locally at high frequency) and at low frequency.

When a genome sequence of a given sample results in an ambiguous nucleotide at a given position, this may be caused by a double infection, but since mutations arise during replication in the host, mixed sequences can also be the result of a quasi-species replicating in an individual (Gregori *et al*. 2021). Before interpreting such data as evidence for recombination, it is essential to evaluate the complete genome, instead of zooming in at a few regions that are polymorphic, as was recently done (Taghizadeh *et al*. 2021). A bioinformatical approach was followed by another group, who analyzed over 304 000 genomic sequences for evidence of recombination using a newly developed software tool (Varabyou *et al*. 2021). Their approach identified 225 genomes as potential resultants of recombination events, but the method was not suitable to define breakpoint locations. In fact, the method seems to report homoplastic events similar to our approach. Whereas those authors conclude these to be the result of recombination events, the candidates they present do not resemble chimeric sequences as one would expect to have arisen in such cases. We differ in our interpretation of the origin of homoplasies: we consider recombinations are relatively unlikely events, although they have occasionally happened. In our opinion, based upon this new analysis, the vast majority of reported homoplasies have arisen from parallel evolution.

## Comparing the most influential VoCs and identification of a ‘missed’ one

There are incentives to define a variant as a VoC when a local outbreak expands, as the virus characteristics, rather than insufficient measures to contain its spread, can be held partly responsible. A claim of a given lineage to represent a novel VoC must at least be backed by experimental evidence to illustrate that higher host titers are reached, or infectivity is increased *in vitro*. However, a given lineage can also expand in a geographic area as a result of a founder effect: if a subpopulation of the virus (belonging to a given lineage) hits a highly susceptible human population in any given area, that lineage will continue to propagate, and its local incidence will increase. This must be considered before calling a rapidly growing subpopulation a VoC. Nevertheless, the Alpha variant was clearly able to outcompete other locally occurring variants in Europe, and later in the United States. In contrast, the spread of Beta and Gamma variants was mainly restricted to Southern Africa and South America, respectively, whereas the Delta variant has turned out to be highly successful on a global scale. However, during the earlier phase of the pandemic, other lineages were highly successful without being nominated a VoC.

**Figure 5.**
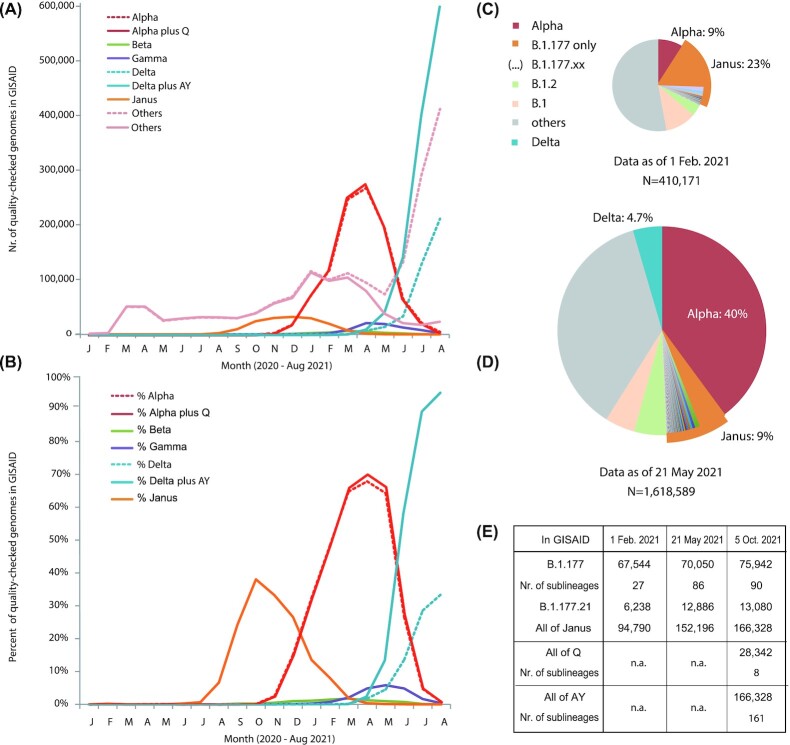
Trends over time for the major VoCs and Janus. Panel **(A)** shows absolute numbers recorded per month, based on data downloaded from GISAID on 5 October 2021. Panel **(B)** shows the same data as fractions of the total, illustrating the waves of Janus, Alpha and Delta. The other VoCs were far less significant on a global scale, based on the submitted genome sequences. Dotted lines in panels (A) and (B) represent numbers for the strictly recorded Alpha and Delta variants, while solid lines represent numbers when variants of Q were added to Alpha and AY variants were added to Delta. Since sequencing and submitting genomes takes time, September 2021 is not included in the graphs. Panels **(C)** and **(D)** show the fractions of lineages belonging to Janus (B.1.177 and the various B.1.177.XX lineages), based on the datasets of 1 February and 21 May, respectively. Panel **(E)** shows how Janus is broken up in various sublineages, whose numbers vary over time, and how offspring of Alpha became variants of Q and that of Delta became multiple variants of AY. n.a.: not applicable.

We zoomed in on the B.1.177 variant and its sublineages, which we now collectively call ‘Janus’ (after the two-faced Roman god), because this subpopulation of SARS-CoV-2 represented a key turning point for the pandemic. In the second half of 2020, Janus was highly successful and spread on a global scale, only to be overtaken by Alpha, the VoC that subsequently had to give way to Delta, as Fig. 5 illustrates. The rapid spread of a variant carrying the S:A222V mutation in Europe mid-2020 (Hodcroft *et al*. 2021) was mainly caused by members of Janus. Although in absolute numbers Janus produced a relatively flat and broad wave (Fig. 5A), its monthly fractions of sequenced genomes produced a clear peak around October 2020, when it reached 40% of all GISAID genomes with collection dates for that month (Fig. 5B). These data are based on a second GISAID download, performed on 5 October 2021. By then, over 3.6 million genomes passed our quality score test, representing a 9-fold increase in eight months. Accumulatively, of these, 29% were Alpha and 4% belonged to Janus. A previous dataset that had been downloaded on 21 May 2021 found that these two lineages comprised over half of all sequences present in GISAID at that time.

Despite its success, Janus was never recognized as a VoC, possibly because the lineage B.1.177 had been split up into multiple sublineages, of which some were also highly successful (Fig. 5C–E). Janus represents a group of lineages all containing S:D614G and NSP12:P323L, the two mutations that boosted the spread of the virus, but other lineages not belonging to Janus contained those two mutations as well, so other auxiliary mutations must be responsible for its success. A number of conserved mutations in Janus are synonymous, but all of its members share S:A222V and N:A220V. Whether either or both of these were responsible for the transient success of Janus can't be assessed by our *in silico* approach. The global wave of infections caused by Janus passed mostly unnoticed. Nevertheless, it did produce a wave that in relative fractions was not less significant than the subsequent wave of Alpha. Janus was just never recognized as being more infectious compared with other lineages.

Janus was replaced by Alpha. Beta and Gamma did not cause significant waves on a global scale. Alpha was subsequently outcompeted by Delta. However, the dotted lines of Fig. 5 suggest that in absolute numbers Delta did not reach the same level as Alpha once did, and genomes of ‘other’ variants appeared to peak in August 2021, whereas the fraction of Delta seemed to level off. This effect isn't real: it is caused by the recent split of Delta into AY sublineages: the offspring of B.1.167.2 was first named B.1.167.2.1 and then renamed lineage AY. Since then, 161 sublineages of AY have been defined. When we add these children of Delta back in, it demonstrates that in absolute numbers Delta is by far the most successful variant of this pandemic, and, in relative fractions of sequenced genomes, it has accounted for over 95% of the sequenced genomes submitted to GISAID in August of 2021 (Fig. 5A and B). The recent split of Alpha children that were renamed Q, with at present eight sublineages, had less of an effect, because the importance of Alpha on a global scale seems to be waning. A serious side effect of splitting lineages representing important variants into numerous sublineages is that it may obscure major trends, just like Janus was not recognized because of its many subvariants. By strictly following Delta or B.1.167.2, it appears this VoC is waning, whereas its offspring are by far dominating the current stage of the pandemic.

After correction for sublineages within Janus, Alpha and Delta, it is obvious that each of these variants resulted in higher fractions of monthly sequenced genomes compared with the previous one (Fig. 5B). This effect may partly be explained by sequencing biases, but it also reflects the continuous adaptation of the virus to its novel human host. During this adaptation, each of these three variants was more successful than the previous one. Nevertheless, their increasing success was not the result of a continuous path of adding novel mutations to an already successful variant. Instead, Janus, Alpha and Delta each contain a unique combination of mutations that is responsible for their increased infectivity. This suggests a degree of cooperativity and co-selectivity for their individual lineage-specific mutations.

## Comparing three widely spread VoCs

We have already elaborated on the observation that recurrent mutations are abundant in various lineages over time. In Table 1, the LCS mutations conserved in >95% of members of Janus, Alpha (including Q) and Delta (including AY) are summarized, with the earliest date of isolation in the GISAID database. Most of these mutations had been observed before, in other lineages (Table 1). Only two mutations arose after April 2020, namely S:P681R and N:D63G.

**Table 1.** Mutations found in >95% of the genomes of high quality belonging to the lineage consensus sequence (LCS) of Janus, Alpha (including Q lineages) and Delta (including AY lineages). Mutations found in all three lineages are shown in bold.

Mutation in:	NT position^a^	AA position^b^	In LCS of Janus	In LCS of Alpha	In LCS of Delta	Earliest date in GISAID^c^	Effect of mutation
noncoding 5′-UTR	g210t	n.a.			yes	n.a.	
noncoding 5′-UTR	**c241t**	**n.a**.	**yes**	**yes**	**yes**	n.a.	
ORF1ab, in NSP1	t445c	syn	yes			n.a.	
ORF1ab, in NSP2	c913t	syn		yes		n.a.	
**ORF1ab, in NSP3**	**c3037t**	**syn**.	**yes**	**yes**	**yes**	n.a.	
ORF1ab, in NSP3	c3267t	NSP3:T183I		yes		Mar 2020	
ORF1ab, in NSP3	c5388a	NSP3:A890D		yes		Mar 2020	
ORF1ab, in NSP3	c5986t	syn.		yes		n.a.	
ORF1ab, in NSP3	c6286t	syn.	yes			n.a.	
ORF1ab, in NSP3	t6954c	NSP3:I1412T		yes		Mar 2020	
ORF1ab in NSP6	del11288–11297	NSP6:S106-/					
G107-/F108-		yes		Feb 2020			
ORF 1b, in NSP12	**c14408t**	**NSP12:P323L**	**yes**	**yes**	**yes**	Jan 2020	Enhances interaction NSP12/NSP8, increases replication rate (Ilmjärv *et al*. 2021)
ORF 1b, in NSP12	c14676t	syn.		yes		n.a.	
ORF 1b, in NSP12	c15279t	syn.		yes		n.a.	
ORF 1b, in NSP12	g15451a	NSP12:G671S			yes	Mar 2020	
ORF 1b, in NSP12	t16176c	syn.		yes		n.a.	
ORF 1b, in NSP13	c16466t	NSP13:P77L			yes	Feb 2020	
noncoding	g21255c	n.a.	yes			n.a.	
S	c21618g	S:T19R			yes	Apr 2020	
S	del21766–21772	S:H69-/V70-		yes		Jan 2020	Enhances infectivity
S	del21991–21994	S:Y144-		yes		Jan 2020	
S	c22227t	S:A222V	yes			Feb 2020	Mutation is used as ‘marker’ for B.1.177 members
S	t22917g	S:L452R			yes	Apr 2020	In RBD, enhances ACE binding (Motozono *et al*. 2021)
S	c22995a	S:T478K			yes	Apr 2020	Immune evasion (Di Giacomo *et al*. 2021)
S	a23063t	S:N501Y		yes		Mar 2020	Enhances ACE binding
S	c23271a	S:A570D		yes		Apr 2020	Affects RBD (Yang *et al*. 2021)
S	**a23403g**	S:**D614G**	**yes**	**yes**	**yes**	Jan 2020	Increases TMPRSS2 and furin sensitivity, improves cell entry (Ilmjärv *et al*. 2021)
S	c23604a	S:P681H		yes		Mar 2020	Increases furin sensitivity (Mohammad, Abubaker and Al-Mulla 2021)
S	c23604g	S:P681R			yes	**Jun 2020**	Increases furin sensitivity (Liu *et al*. 2021) and fusogenicity (Saito *et al*. 2021)
S	c23709t	S:T716I		yes		Mar 2020	Affects ACE/TMPRSS2 interaction
S	g24410a	S:D950N			yes	Mar 2020	
S	t24506g	S:S982A		yes		Mar 2020	Stabilizes S with D614G and A570D (Ostrov 2021)
S	g24914c	S:D1118H		yes		Mar 2020	
ORF3a	c25469t	ORF3a:S26L			yes	Mar 2020	
M	t26767c	M:I82T			yes	Mar 2020	
M	c26801g	syn.	yes			n.a.	
ORF7a	t27638c	syn.			yes	n.a.	
ORF7a	c27752t	syn.			yes	n.a.	
ORF8	c27972t	ORF8:Q27*		yes		Mar 2020	
ORF8	g28048t	ORF8:(R52I)^d^		yes		Mar 2020	
ORF8	a28111g	ORF8:(Y73C)^e^		yes		Mar 2020	
ORF8	del28248-28253	ORF8:D119I, ORF8:F120del			yes	n.a.^f^	
N	g28280c, a28281t, t28282a	N:D3L		yes		Mar 2020	
N	a28461g	N:D63G			yes	**Jun 2020**	
N	g28881a, g28882a, g28883c	N:R203K/G204R		yes		Jan 2020	Suggested to have arisen from intra-genomic recombination (Leary *et al*. 2021)
N	g28881t	N:R203M			yes	Mar 2020	
N	c28932t	N:A220V	yes			Feb 2020	increased stability of N (Rubayet Ul Alam *et al*. 2021)
N	c28977t	N:S235F		yes		Jan 2020	
N	g29402t	N:D377Y			yes	Feb 2020	
ORF10	g29645t	ORF10:V30L	yes			n.a.^f^	

n.a.: not applicable; syn.: synonymous.

a Nucleotide position is given with reference to the reference sequence NC_045512.2.

b Amino acid position is given for the final protein product.

c Earliest isolation dates of genomes assigned to Alpha or Delta prior to June 2020 were ignored.

d Since this mutation is downstream of an introduced stop-codon, it is not translated, but might have ncRNA or genomic structure effects.

e The deleted amino acids in ORF8 could not be identified in GISAID.

f Mutations in ORF10 are not captured in GISAID.

To our surprise, there were numerous entries in GISAID of Alpha or Delta genomes isolated as early as March 2020, months before these lineages were discovered in the UK and India, respectively. At the time of writing, 38 Alpha genomes in GISAID were sequenced from samples recorded to have been isolated prior to 1 May 2020, and 106 genomes were from Delta isolated in 2020. These early entries were originating from various countries and continents. If those variants had arisen and spread globally that early during the pandemic, the question is why they had not expanded in numbers sooner, given their increased infectivity. The anomaly cannot be explained, as a lag phase of over a year seems too long for the highly infective Delta. For the dates recorded in Table 1, we extracted the earliest observed mutation when discovered in a lineage other than in the VoC.

Mutations in the spike protein are of course important for the success of a VoC. As an example, S:N501Y is critical for Alpha, although Delta can manage without it. Increased binding dynamics of S with N501Y have been experimentally confirmed (Starr *et al*. 2020; Chan *et al*. 2021). However, the same S:N501Y substitution had arisen in SARS-CoV-2 on multiple occasions, as early as March 2020, yet it has not resulted in discernible increased infectiousness. Its presence only started to increase in genomes sequenced from October 2020 onward, when Alpha was flourishing. Leung and colleagues listed 18 mutations present in a representative of B.1.1.7 that were not also present in the Welsh 501Y variant that lacked apparent increased infectivity (Leung *et al*. 2020). Presumably, for higher competitiveness, or perhaps to compensate for such a change, other mutations in addition to S:N501Y need to be present in an Alpha background.

With the early discovery dates of the listed mutations, Table 1 once more illustrates the scale of recurrent mutations. Recently, *in vitro* experiments were described showing that mutations, selected for remdesivir drug resistance, indeed frequently resembled mutations seen in the natural populations, again suggesting that the repertoire of the virus is limited. It should be noted that these mutations were observed without any form of immune selection (Szemiel *et al*. 2021). Table 1 further implies that mutations present in a VoC were previously not sufficient to create a globally successful variant unless it was introduced into the right genetic background. For a few mutations, their combined effect is obvious, for instance for S:D614G combined with NSP12:P323L. Strains with these two mutations have taken over on a global scale, although the individual presence of these two mutations did not produce successful lineages (Ilmjärv *et al*. 2021). Nevertheless, it was estimated that the fitness benefit of these two mutations in combination was very minor, when comparing pre-September 2020 US data with later data (Kepler, Hamins-Puertolas and Rsmussen 2021). These authors speculate that their counter-intuitive finding may be due to genetic backgrounds in which these mutations were found, but by September 2020 Janus was already on the rise (Fig. 5B) and this may have obscured the analysis by Kepler*et al*. somewhat.

Two independent mutations that create an advantageous effect at a conserved position, but with different amino acid substitutions, are S:P681H and S:P681R, occurring in Alpha and Delta, respectively (Liu *et al*. 2021; Mohammad, Abubaker and Al-Mulla 2021). Both were created by a change of the nucleotide at c23604 (to 23604a in Alpha and to 23604g in Delta). A synthetic construct of a Delta genome with the complete spike protein from Alpha displayed decreased fitness toward human cells *in vitro* compared with Delta, and compared with Alpha, suggesting that the spike protein of Delta is better equipped for its own genetic background than the spike of Alpha is (Liu *et al*. 2021). The role of S:P681R in enhanced furin sensitivity is currently being debated (Zhang *et al*. 2021) but the mutation does lead to a higher fusogenicity (Saito *et al*. 2021; Zhang *et al*. 2021) that contributes to higher infectivity. Another amino acid that is also mutated in two ways is S:R203, changing to R203K (together with G204R) in Alpha, and to R203M in Delta. As mentioned, this mutation hotspot may be caused by intra-genomic recombinations (Leary *et al*. 2021). It has been proposed that mutations at position 203 in N affect its affinity to the viral genome during capsid packaging, for which the c241t mutation in the 5′-UTR, that arose very early on, may be beneficial as well (Rubayet Ul Alam *et al*. 2021). Mutations in this region of N have been linked to more efficient genome replication and packaging (Syed *et al*. 2021).

The improved spread of Alpha and Delta is most likely the result of a combination of factors. Recently, the theoretical factors essential for aerosol spread of respiratory viruses were summarized as follows: causing an asymptomatic but contagious phase of infection, high viral loads, virus stability in air and sufficient binding affinity to human cells (Lee 2021). Aerosol spread has been demonstrated over a distance of 2 m in a hamster cage study for Alpha (Port *et al*. 2021). Delta seems to result in higher viral loads, increasing its ability to spread via aerosols.

## What do these observations mean for the evolution of SARS-CoV-2?

Purifying selection has been shown to be a major driver of the evolution of this virus (Rochman *et al*. 2021; Singh and Yi 2021; Tonkin-Hill *et al*. 2021) although for some sites positive selection has also been demonstrated (Rochman *et al*. 2021). RNA deamination may be at the basis of an observed overrepresentation of C to T transition and G to T transversion events observed in intra-host diversity (Tonkin-Hill *et al*. 2021), as well as in global genome comparison (Gregori *et al*. 2021; Rochman *et al*. 2021). Those mutations were more often the result of RNA damage and editing than replication errors (Tonkin-Hill *et al*. 2021). It was recently reported that triplets with a cytosine in the middle (NCN) are more often subject to mutation (Rochman *et al*. 2021). It was speculated by those authors that over time the relatively high dN/dS ratio (>1) might decrease because of negative selection, especially if corrected for a mutational bias toward NCN. Negative selection is unlikely to result in homoplasies, but positive selection may very well cause fixation of the same mutation arising from independent events. Rochman and colleagues described that >100 nonsynonymous substitutions appeared to have emerged multiple times; we independently found even more recurrent substitutions when including nonsynonymous mutations. Those authors initiated the highly relevant search to identify possible linked (co-selected) mutations, as a result of epistatic interactions and identified a central hub for S:D614G and for N:R203K/G204R. However, their dataset was pre-Delta and was biased toward Alpha, and to a lesser extent Beta and Gamma. It seems that Delta, the most successful lineage to spread more rapidly in humans so far, is the result of an alternative evolutionary route, compared with the other VOC.

Remaining key questions are as follows: which of its many mutations make a particular VoC more highly successful, in terms of population dominance? A high variation rate in genomes belonging to Delta has been described before (Suratekar *et al*. 2021), but in which direction is the VoC evolving? Although theoretically possible, is it likely that a given VoC will independently evolve to collect mutations similar to those that resulted in increased fitness in previous VoC, thus creating an ever more ‘successful’ VoC?

**Figure 6.**
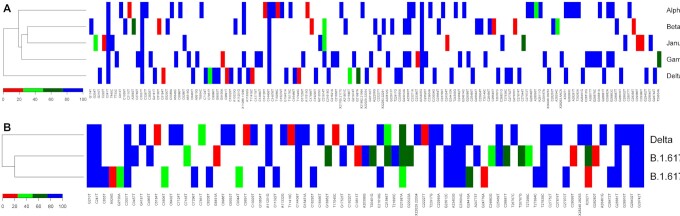
Matrix showing mutations accumulating in >10% of the members of selected lineages, based on the dataset of 5 October 2021. Panel **(A)** shows the VoCs Alpha (with Q included), Beta, Gamma, Delta (with AY included) and the group of Janus. Mutations that were conserved in >80% of their members are shown in blue (note that this degree of conservation is more relaxed than >95% that was used for their LCS). The cladogram to the left shows that Delta is less related to the other four lineages. Panel **(B)** compares the mutation frequency of Delta (plus AY) with that of two related lineages that are not widely spreading. Mutations present in the LCS (at 95%) of Delta plus AY but absent in the other two are indicated by arrows, with grey for mutations that have been noted in earlier, unrelated Pango lineages. The black arrow points to N:D63G that arose in June 2020. Two mutations occurring at the same position are boxed.

To address these key questions, we compared all genomes belonging to the lineages Alpha/Q, Beta, Gamma, Delta/AY and Janus (Fig. 6). This analysis clearly demonstrated that Delta shares fewer mutations with Janus, Alpha, Beta and Gamma than do any two of them, as indicated by the cladogram to the left of the matrix in panel A. Mutations shown in red were conserved in >75% of the genomes; those shown in the other colors have evolved since the founder of that lineage arose: red is present in at least 10% of the genomes, light green in at least 25% and dark green in at least 50%. These post-founder mutations are indicative of the direction in which these VoCs are evolving.

There were only five mutations recorded in members of Alpha-plus-Q that did not reach the 75% cutoff, eight in Beta, three in Gamma and eight in Delta. Since the datasets vary enormously in size, these numbers cannot directly be compared. Due to the wide spread of Delta, it has had ample opportunity to mutate further. Nevertheless, although we added over 166 000 AY genomes, there are not that many novel mutations reaching 10%, because of the overwhelming number of Delta itself. None of those novel mutations arising in Delta were observed in the other VoC or in Janus, and none of them are located within the highly evolving regions of the other SARS-CoV-2 genomes shown in Fig. 1. This could be suggestive of novel fitness direction the Delta variant is now pursuing, directed by one or more powerful new mutations.

We have wondered why Delta (Pango lineage B.1.617.2) was so successful in its spread, whereas two related lineages (B.1.617.1 and B.617.3) were not. Upon comparison of the three (Fig. 6B), we identified 18 mutational events unique to Delta and present in >75% of its members. Mutations unique to the two less competitive strains, but absent in Delta, were less relevant, although it is possible they were attenuating. The already mentioned mutation at nucleotide 23604 resulting in S:P681H in Alpha and S:P681R in Delta is also found as S:P681R in the less successful variants B.1.617.1 and B.1.6173. One Delta-unique mutation, c22995a, giving S:T478K, had not only been observed in a nonrelated variant in April 2020 (Table 1) but also had previously changed to c22995t to give S:T478I in an unrelated, unsuccessful lineage. The Delta-unique mutation at position t26767c, to give M:I82T, was alternatively changed to a G to give M:I82S in B.617.1, as indicated by a box in the figure. Although it can't be excluded that the nature of the amino acid change is partly responsible for strengthened fitness phenotypic effects in Delta, we consider these less likely to be behind the success of Delta.

All other Delta-unique mutations (except for one) have been found in earlier variants isolated prior to April 2020 (Table 1). That leaves mutation a28461g in N, giving N:D63G, as highly interesting, as it arose relatively late in the pandemic—it was first detected in June 2020. The change in N is most likely to have affected infectious virus titers, as the nucleoprotein N is the most abundant protein produced by this virus and is responsible for the packaging of the viral genome. We speculate that genome packaging may be more efficient with the mutated Delta nucleoprotein. This might contribute to higher infectious particle titers, and subsequently more effective aerosol transmission (Lee 2021).

As the SARS-CoV-2 virus has evolved to better adapt to its new human host, it has accumulated numerous mutations, some essential, others merely hitchhikers/passengers. The RNA genome of this virus has incredibly intricate folding signals built into its primary sequences that affects the secondary and tertiary structures required for efficient sequential folding, transcription regulation and genomic packaging. Mutations that weaken these structures would quickly reduce packaging efficiency, reducing infectious virus titers and increasing defective interfering particle production, while mutations increasing the speed of this process would provide a clear advantage to the virus.

Once the virus has entered a host cell, it is largely this aspect of secondary genomic structure that governs genome packaging, combined with transcription regulation that is equally critical. Structural packaging constraints of the primary genomic sequence may be severely limiting the repertoire of mutations that SARS-CoV-2 can withstand at any given moment without very precise compensatory changes elsewhere to correct any introduced RNA-folding structural changes. Alternatively, critical changes in the N protein and in the replication transcription complex (RTC) might be needed to accommodate any new genomic structures.

Although small changes in N gene expression that improve its production could be beneficial, it is more likely that changes in its many other chaperone functions are having significant effects on the efficiency of virus production, resulting in vastly higher virus titers, such as the thousand-fold increase recently observed for the Delta strain (Li *et al*. 2021). In addition to packaging the genome, N is known to associate with and stabilize the RTC, potentially mediating its efficiency as well. This illustrates that the focus on the functions of S that has dominated the scientific discourse may result in missing clues to Delta's success. However, the importance of N:D63G needs to be assessed by modeling and by actual wet-lab evidence. Although the significance of this mutation might not be clear, D63G is in the first of three disordered domains of the nucleoprotein. The SARS-CoV-2 nucleocapsid protein is dynamic, disordered, and phase separates with RNA (Cubuk *et al*. 2021), and can generate ‘membraneless organelles’ to localize the viral RNA genome using liquid–liquid phase separation (Savastano *et al*. 2020; Scoca and Di Nunzio 2021). In a way, the nucleoprotein acts as a chromatin condensing agent, like histone proteins do in eukaryotic cells. Mutations in histone proteins can strongly increase genome replication and such mutations are frequently implemented in the onset of cancer. Considering this, it is maybe no surprise that slight modifications in the nucleoprotein of SARS-CoV-2 can have large effects on its rate of multiplication and the titers it can produce.

By zooming in on the individual mutations of each of these VoC lineages, it becomes clear that very few of their mutations were unique to those lineages; instead, many of them have been observed in previous SARS-CoV-2 genomes, in variable combinations. It seems that the mutational repertoire of SARS-CoV-2 is relatively limited by what is ‘needed to adapt’ and what is ‘allowable within the complexities of the virus itself’ and how it interacts with its current host. This can be solved in alternative ways, as is illustrated by the three waves caused by Janus, Alpha and Delta. The mutations that they accumulated must individually or in combination be responsible for their increased ability to spread in human populations. However, what works for one VoC does not necessarily work for others.

The above does not necessarily explain why the progenitor to Alpha collected a sudden burst of many additional mutations. The founder of Alpha has evolved over a relatively short period of time, collecting multiple mutations that became fixed in its offspring (Farkas *et al*. 2020; Gómez-Carballa *et al*. 2020). Likewise, Delta appeared to have undergone a burst of mutations. On the one hand, this is in stark contrast to the overall low mutation rate that is the hallmark of the family of coronaviruses in general, to which SARS-CoV-2 is no exception. The mutation range of SARS-CoV-2 has been estimated as 2 mutations per month, or 10^−3^ mutations per site per year (Candido *et al*. 2020; Fauver *et al*. 2020). This is within the range of mutation rates for other positive-strand RNA virus species such as Hepatitis C virus (summarized in Wassenaar *et al*. 2020) but lower than that of Foot-and-Mouth Disease virus (around 10^–2^ ssy, Biswal *et al*. 2019) and higher than that of West Nile virus isolated from humans (10^–4^ ssy; Añez *et al*. 2013). However, since SARS-CoV-2 has spread much more rapidly and infected far more people than these other virus species, it has undergone many more replication events in a single year, so that the mutation rate of SARS-CoV-2 per replication event is significantly lower than that of other single-strand positive RNA virus families. This is most likely because, like all other coronavirus species, it performs high-fidelity proofreading during replication of its unusually large and complex RNA genome (Robson *et al*. 2020; Romano *et al*. 2020), which occurs in the multi-protein RNA-dependent RNA polymerase complex, also known as the RTC.

The RNA proofreading by the SARS-CoV-2 RTC is mainly performed by the bifunctional enzyme NSP14. This protein also has the N7-methyltransferase activity that is responsible for methylation of the guanylate end-cap of the viral genome. Most importantly to this discussion, NSP14 has 3′–5′ exoribonuclease activity responsible for the removal of faulty RNA products (Ogando *et al*. 2020). Mutations in NSP14 can diminish or abolish proofreading during replication of the RNA genome, resulting in a higher mutation rate, as mistakes introduced during genome replication are at some point no longer removable (Ogando *et al*. 2020). However, no mutations in NSP14 were consistently observed or conserved in the VoC lineages discussed here, suggesting that, overall, genomic fidelity is extremely critical to long-term viral fitness.

The P323L mutation in NSP12 could, in theory, be held accountable for sloppier replication, and its presence was associated with an increased mutation rate compared with the consensus of the progenitor genome originating from Wuhan (Jiang, Yin and Xu 2020). That NSP12:P323L mutation has been around since February 2020, and so it is entirely possible that observation bias led to the conclusions drawn by Jiang and colleagues. It is possible that a ‘mutator phenotype’ is transiently helpful for acquiring a burst of new fitness mutations—but is then highly detrimental to long-term viral fitness, as discussed above. Theoretically, compensatory correcting mutations could occur elsewhere within the many other proteins of the RTC to return the mutation frequency to a more acceptable level. However, an equally likely explanation for ‘sporadically enhanced’ mutation rates may be the medical use of nucleotide analogs as therapeutic drugs that target the viral replication machinery, when used in immunocompromised individuals suffering from a prolonged infection (weeks and months) with ongoing low-level viral replication. These drugs are known to induce drug-resistant ‘treatment escapees’ with a high number of mutations.

There is some evidence that immune-compromised individuals being treated with nucleotide-analog therapeutic drugs are at increased risk for generating virus mutants. In the first reported case, there was a failed attempt to rescue a long-term immune-suppressed Covid-19 patient using remdesivir and convalescent plasma (Kemp *et al*. 2021). Similarly, treatment of long-term Covid infections with monoclonal antibodies has also selected for immune evasive mutants, in one case resulting in the powerful E484K mutation (Jensen *et al*. 2021). Thus, both immune evasion and resistance to remdesivir and other antiviral therapeutics can result in the direct creation and selection of mutants, a side effect that has been warned about (Colson *et al*. 2021). Here, we point out that the resultant mutations are often the same as have been observed at early times in the pandemic. As Mari and colleagues have pointed out, mutations in RdRp selected for by antiviral treatments are under purifying selection and have so far not caused any identified escape mutations resulting in global expansion (Mari *et al*. 2021). Whether the sudden rise of Alpha, Delta and Omicron, all of which have collected many mutations for which intermediate ancestors have not been identified, were the result of antiviral or antibody therapy can at present not be determined.

Part of the extraordinary and unexpected limited repertoire of mutations reported here for SARS-CoV-2 may be due to immune selection, which is one factor at play to ‘fix’ mutations in the viral population. Forni *et al*. (2020) report that mutations for S and N proteins were more common in the B-cell/antibody-recognized epitopes than in non-epitope (non-antigenic) positions, while epitopes recognized by CD4^+^ and CD8^+^ T cells had the same level of variability as non-epitope positions (Forni *et al*. 2020). This makes sense, since mutational pressure to escape antibodies targeting them would be the greatest for these two surface-exposed viral proteins as the mature viral particles are released into the intercellular antibody-rich milieu from host cells, and the strongest epitopes are usually formed by the tertiary structures of intact proteins. T cell epitopes, on the other hand, are determined by secondary structures of small fragments of viral proteins displayed by MHC I and III complexes on the surfaces of any infected cells and by phagocytic dendritic cells that have engaged with them. T cells specifically target the infected cells, not the released individual virus particles, so the pressure to escape them would be on viral proteins most involved in MHC-antigen-presentation pathways. In addition to the commonly targeted spike protein, the *ORF3a* and *ORF8* genes produce proteins that are highly antigenic (Forni *et al*. 2020; Flower *et al*. 2021). These proteins are secreted by infected cells into the external environment during viral replication, but the same proteins are completely absent in the mature virus particle, so there is no mutational immune pressure on them for success in the cellular release, person-to-person transmission or the infection of new cells in the same host.

A limitation to our analytical approach is that the lineage assignments to which the deposited genomes were assessed are not stable, as they are regularly updated and sometimes redefined at GISAID. This is problematic as it hampers comparison of historical data, although we acknowledge that reshuffling of genomes is sometimes advisable, based on advanced insights. A second weakness of using the defined lineages is that, as we observed here, some lineages are very finely subdivided, to the point that a single mutation defines a new lineage (as for the B.177.XX lineages within Janus), while other lineages are less clearly defined and more heterogeneous (as was B.1, and now Delta). Moreover, the nomenclature can be confusing, as the relationship of various lineages cannot always be inferred from their names. Alternative methods to describe subpopulations of this virus exist, notably the clades that depend on the presence or absence of telltale mutations (Hadfield *et al*. 2018; Mercatelli and Giorgi 2020). This is also risky, when such mutations evolve on multiple independent occasions, as it results in combining genomes that clearly have different phylogenetic relationships.

The problems of nomenclature and lineage assignments are not easy to solve, but at least an increased awareness by the wider scientific community that SARS-CoV-2 undergoes parallel evolution on a significant scale would be helpful in better framing this discussion.

## Conclusions: how can knowledge of the first three waves help with the omicron wave?

At the time of writing (December 2021) there is extensive news coverage about the newly emerging Omicron VoC, which appears to be spreading across the globe even more quickly than Delta, despite a significant increase over time in the percentage of vaccinated people. Initial reports regarding this VoC mentioned that it is ‘heavily mutated’ in the spike protein, and thus more likely to be immuno-evasive. There has been much concern that Omicron will overwhelm countries around the world once again. A quick look at the Omicron genomes so far submitted does indeed find many mutations within the Spike protein, but most, if not all, of these spike mutations have been recorded in previous lineages. Furthermore, the number of mutations in Spike that are consistently conserved in Omicron appear to be far fewer than initially reported.

Of the 6 045 431 SARS-CoV-2 genomes found in GISAID on 12 December 2021, there were 3365 Omicron genomes, most of which are incomplete (only 35 are ‘high-coverage’, and only 18 had no gaps or missing sequences annotated). The number of reported mutations in the Omicron spike protein is reported as ‘more than 30 mutations’ (Karim and Karim 2021). However, there are a few points to be made here: first, individual genome sequences can often be messy, as evidenced by <1% of the Omicron genomes being of good quality (18/3365 = 0.5%). Second, variability in a noisy dataset can lead to problems when the sample size is small. Third, a multi-nucleotide deletion event can affect multiple adjacent amino acids; in total, 21 of the 35 reported amino acid changes in the Omicron proteome are due to only eight deletion events. Lastly, most of the mutations consistently reported in the Omicron genomes have been observed in other lineages before. Since Omicron lacks many mutations that are conserved in Alpha, it is unlikely that Omicron evolved from an Alpha member.

One important implication of our findings is that if SARS-CoV-2 indeed has a limited repertoire of allowable mutations, its repertoire is also limited in terms of the individual overlapping complexities of the functions of its proteins and their antigenicity during different stages of virus infection and propagation. Instead of the frightening theoretical possibility of this virus producing millions of different epitopes that need to be therapeutically anticipated during vaccine development, we may now be able to predict a very limited subset of ‘probable’ epitopes (both linear and structure-dependent) by modeling its antigenic proteins with novel combinations of mutations that have already been observed. This opens a new and effective strategy for the development of future vaccines and therapeutic drug development.

## Funding

This work was supported in part by NIH/NIGMS grants 1P20GM121293 and UL1 TR003107, by a National Science Foundation award (no. OIA-1946391) and by funding from the Arkansas Research Alliance.

## Data availability

The main matrix shown in Fig. 2 can be made available as a .cvs file upon request.

## Supplementary Material

fuac003_Supplemental_File
