# Supplementary material for: The first three waves of the Covid-19 pandemic hint at a limited genetic repertoire for SARS-CoV-2
Source: FEMS Microbiol Rev. 2022 Jan 24;46(3):fuac003. doi: 10.1093/femsre/fuac003 (PMC9075578; doi:10.1093/femsre/fuac003)
Supplement: fuac003_Supplemental_File [file fuac003_supplemental_file.docx]

**Supplementary Tables and Figures**

**Table S1.** Incomplete conservation of four mutations that evolved early in the pandemic suggest that reversions occurred.

|  | **Percentage of Lineage members with the mutation** | | | |
| --- | --- | --- | --- | --- |
| **Lineage** | **c241t** | **c3037t** | **NSP12:P323L** | **S:D614G** |
| A.10 | 0 | 38 | 0 | 100 |
| B.1.1.161 | 91 | 78 | 86 | 14 |
| B.1.456 | 83 | 8 | 100 | 100 |
| B.1.260 | 99 | 88 | 99 | 52 |
| B.1.184 | 100 | 100 | 53 | 100 |
| B.1.411 | 57 | 76 | 71 | 100 |
| B.1.1.96 | 100 | 100 | 100 | 69 |
| B.1.473 | 95 | 95 | 95 | 89 |
| B.1.113 | 97 | 100 | 78 | 100 |
| B.1.447 | 100 | 100 | 100 | 75 |
| B.1.95 | 80 | 92 | 96 | 100 |
| B.1.1.175 | 100 | 100 | 78 | 100 |
| B.1.405 | 89 | 94 | 100 | 100 |
| B.1.421 | 86 | 100 | 100 | 100 |
| B.1.1.35 | 100 | 94 | 94 | 100 |
| B.1.393 | 59 | 100 | 100 | 100 |
| B.1.449 | 100 | 100 | 100 | 88 |
| B.1.1.290 | 90 | 100 | 55 | 100 |
| B.1.379 | 94 | 97 | 97 | 100 |
| B.1.1.291 | 97 | 96 | 94 | 99 |
| B.1.36.19 | 70 | 100 | 100 | 100 |
| B.1.484 | 94 | 94 | 100 | 100 |

**Table S2.** Homoplasies identified in lineages containing >1000 genomes and in deletions for the complete dataset

| **Substitution** | **c18877t in NSP14** | **c22444t in S** | **c26735t in M** | **c28854t N:S194L** |
| --- | --- | --- | --- | --- |
| B.1.240  B.1.243 | 0  0 | 0  0 | 0  0 | 98.6%  99.5% |
| B.1.36  B.1.36.17 | 94.3%  100% | 94.1%  94.0% | 99.3  99.9 | 97.1%  99.7% |
| B1.160 | 99.8% | 0 | 98.3 | 0 |
| **Substitution** | **a1163t in NSP2:I20F** | **g19542t in NSP14** | **c19718t in NSP15** |  |
| D.2 | 99.9% | 0 | 0 |  |
| B.1.1.315 | 99.7% | 95.4% | 99.7% |  |
| B.1.1.70 | 0 | 0 | 91.0% |  |
| **Deletions** | **del 11288-11297**  **NSP3:S106-/G107-/F198-** | **del 21766-21772**  **S:H69-/V70-** |  |  |
| P.1 (Gamma) | 100% | 0 |  |  |
| B.1.351 (Beta) | 86.1% | 0 |  |  |
| B.1.1.7 (Alpha) | 99.6% | 99.5% |  |  |
| B.2.258 group  B.1.388  B.1.375  B.1.1.298 | 0  0  0  0 | >93%  100%  92.4%  73.5% |  |  |

**Supplementary Figure**

**Figure S1**. Homoplasies in sublineages of B.1.177. A total of 11 sequence fragments of the Sars-CoV-2 genome are shown that contain a mutation in at least one sublineage of B.1.177. The mutations are indicated in green. B.177 is shown at the top, and B177.16 and B.1.177.23, which do not contain mutations in the shown regions, are included for completion. Two homoplasies are visible in the red square. The presence of g8872c in NSP4, g10870t in NSP5 and S:A262S in members of B.1.177.10, B.1.177.9, B.1.177.4 and B.177.1 can’t be explained without allowing for parallel evolution, reversion or recombination.
